# Supplementary material for: Robust proportional overlapping analysis for feature selection in binary classification within functional genomic experiments
Source: PeerJ Comput Sci. 2021 Jun 1;7:e562. doi: 10.7717/peerj-cs.562 (PMC8176540; doi:10.7717/peerj-cs.562)
Supplement: Supplemental Information 1 — Brier score, error rate and sensitivity metrics for the benchmark problems considered in the article. [file peerj-cs-07-562-s001.pdf]

SUPPLEMENTAL RESULTS

This section provides the results of remaining datasets for the random partioning of the data i.e. (70%) training, (30%) testing and (30%) training, (70%) testing for all the gene selection and classification methods given in the paper. Table 1, gives the results for the dataset “Tumorc”, when the data is partioned into 30% training and 70% testing parts, it is clear from the table that the proposed method (RPOS) outperforms the rest of the methods for various number of genes on all the classifiers. Table 2, gives the results for the dataset “Breast ”, when the data is partioned into 30% training and 70% testing parts, it is evident from the table that the proposed method (RPOS) is winning in almost in all the scenarios. Similarly Table 3, gives the results for the dataset “Srbct”, for the partioning of data into 30% training and 70% testing parts, it can be seen from the table that the proposed method (RPOS) gives the best results whereas POS is the second best among all the other methods. Similar coclusion could be drawn for the rest of the datasets under various scenarios from the results given in Tables 4-15.

Table 1. Classification error rate, sensitivity and Brier score produced by Random Forest, k Nearest Neighbors and Support Vector Machine classifiers on TumorC dataset when the data is partioned into (30%) training and (70%) testing parts.

		RF						kNN						SVM					
Genes		POS	RPOS	GClust	sigF	Wilc	mRmR	POS	RPOS	GClust	sigF	Wilc	mRmR	POS	RPOS	GClust	sigF	Wilc	mRmR
	Err	0.432	**0.359**	0.408	0.339	0.419	0.451	0.401	**0.390**	0.429	0.421	0.407	0.427	0.481	0.393	0.404	0.483	**0.385**	0.408
5	BS	0.020	**0.017**	0.045	0.331	0.264	0.280	0.025	**0.019**	0.211	0.267	0.262	0.270	0.035	**0.021**	0.233	0.264	0.249	0.251
	sen	0.263	0.357	0.273	**0.361**	0.323	0.299	0.237	**0.419**	0.365	0.350	0.295	0.287	0.386	**0.505**	0.432	0.398	0.103	0.152
	Err	0.444	**0.340**	0.384	0.336	0.389	0.458	0.439	**0.353**	0.432	0.455	0.407	0.420	0.499	0.381	0.438	0.475	**0.356**	0.396
10	BS	0.017	**0.015**	0.048	0.332	0.250	0.278	0.024	**0.017**	0.289	0.282	0.264	0.272	0.032	**0.019**	0.260	0.268	0.245	0.250
	sen	0.221	**0.360**	0.326	**0.364**	0.270	0.252	0.221	**0.362**	0.355	0.331	0.288	0.127	0.332	**0.437**	0.383	0.361	0.094	0.118
	Err	0.429	**0.348**	0.416	0.333	0.381	0.435	0.443	**0.383**	0.404	0.440	0.425	0.422	0.479	0.386	0.433	0.455	**0.377**	0.388
15	BS	0.020	**0.018**	0.055	0.330	0.251	0.267	0.029	**0.024**	0.266	0.282	0.265	0.269	0.039	**0.023**	0.253	0.262	0.254	0.251
	sen	0.198	0.340	0.303	**0.365**	0.264	0.170	0.249	**0.381**	0.313	0.242	0.304	0.275	0.278	**0.436**	0.269	0.344	0.126	0.072
	Err	0.394	**0.334**	0.388	0.330	0.391	0.416	0.446	**0.377**	0.444	0.438	0.419	0.437	0.462	0.354	0.378	0.424	**0.369**	0.396
20	BS	0.014	**0.012**	0.050	0.329	0.249	0.259	0.028	**0.019**	0.293	0.277	0.265	0.276	0.022	**0.014**	0.244	0.259	0.251	0.250
	sen	0.265	0.357	0.333	**0.366**	0.282	0.220	0.339	**0.372**	0.329	0.263	0.311	0.323	0.355	**0.463**	0.346	0.347	0.064	0.096
	Err	0.391	**0.343**	0.379	0.324	0.397	0.430	0.461	**0.380**	0.411	0.449	0.427	0.417	0.462	**0.361**	0.390	0.418	0.386	0.402
25	BS	0.019	**0.017**	0.043	0.328	0.248	0.264	0.032	**0.021**	0.268	0.283	0.267	0.263	0.029	**0.022**	0.238	0.262	0.250	0.256
	sen	0.259	0.366	0.333	**0.370**	0.229	0.235	0.330	**0.369**	0.349	0.231	0.296	0.359	0.314	**0.481**	0.358	0.338	0.080	0.105
	Err	0.405	**0.335**	0.366	0.324	0.409	0.410	0.464	**0.350**	0.443	0.450	0.412	0.430	0.452	**0.357**	0.429	0.428	0.372	0.394
30	BS	0.020	**0.018**	0.041	0.328	0.256	0.256	0.041	**0.025**	0.222	0.280	0.261	0.268	0.031	**0.023**	0.249	0.262	0.253	0.264
	sen	0.256	0.367	0.313	**0.370**	0.193	0.254	0.388	**0.389**	0.319	0.240	0.309	0.327	0.331	**0.460**	0.386	0.314	0.036	0.136

Table 2. Classification error rate, sensitivity and Brier score produced by Random Forest, k Nearest Neighbors and Support Vector Machine classifiers on Breastcancer dataset when the data is partioned into (30%) training and (70%) testing parts.

		RF						KNN						SVM					
Genes		POS	RPOS	GClust	sigF	Wilc	mRmR	POS	RPOS	GClust	sigF	Wilc	mRmR	POS	RPOS	GClust	sigF	Wilc	mRmR
	Err	0.382	**0.359**	0.374	0.469	0.419	0.452	0.340	**0.316**	0.429	0.454	0.432	0.432	0.381	**0.375**	0.419	0.487	0.428	0.428
5	BS	0.015	**0.014**	0.181	0.279	0.285	0.274	**0.014**	**0.014**	0.213	0.277	0.281	0.276	0.017	**0.016**	0.209	0.278	0.302	0.269
	sen	0.678	**0.691**	0.609	0.632	0.677	0.658	0.708	**0.739**	**0.610**	0.701	0.725	0.730	0.643	0.654	0.688	0.590	0.677	**0.742**
	Err	0.359	**0.339**	0.385	0.497	0.400	0.442	0.331	**0.297**	0.340	0.472	0.431	0.427	0.361	**0.345**	0.350	0.515	0.405	0.450
10	BS	**0.011**	**0.011**	0.186	0.276	0.255	0.260	0.014	**0.012**	0.225	0.282	0.273	0.272	0.018	**0.014**	0.230	0.275	0.279	0.270
	sen	0.723	**0.744**	0.612	0.634	0.694	0.675	0.698	**0.787**	0.618	0.691	0.759	0.735	0.674	0.695	**0.788**	0.574	0.676	0.739
	Err	0.345	**0.327**	0.343	0.503	0.409	0.391	0.336	**0.275**	0.294	0.462	0.415	0.433	0.377	**0.322**	0.385	0.506	0.414	0.437
15	BS	**0.012**	0.013	0.165	0.273	0.253	0.240	0.015	**0.014**	0.202	0.281	0.272	0.276	0.022	**0.016**	0.185	0.278	0.281	0.267
	sen	**0.758**	0.748	0.639	0.668	0.695	0.730	0.731	**0.799**	0.664	0.729	0.741	0.718	0.669	0.714	0.623	0.585	0.690	**0.751**
	Err	0.358	**0.303**	0.341	0.487	0.400	0.495	0.324	**0.279**	0.300	0.471	0.437	0.427	0.358	**0.315**	0.386	0.497	0.400	0.479
20	BS	0.011	**0.010**	0.172	0.270	0.249	0.277	0.015	**0.012**	0.203	0.284	0.287	0.273	0.018	**0.012**	0.190	0.280	0.278	0.277
	sen	0.746	**0.764**	0.626	0.694	0.749	0.637	0.720	**0.797**	0.697	0.673	0.687	0.721	0.684	0.723	0.695	0.593	**0.744**	0.727
	Err	0.360	**0.317**	0.347	0.483	0.392	0.454	0.321	**0.298**	0.307	0.435	0.440	0.429	0.372	**0.331**	0.386	0.482	0.397	0.459
25	BS	0.013	**0.012**	0.170	0.266	0.246	0.260	0.015	**0.014**	0.203	0.268	0.280	0.273	0.021	**0.016**	0.181	0.281	0.274	0.270
	sen	0.730	0.755	0.637	0.692	**0.758**	0.680	0.749	**0.796**	0.680	0.722	0.720	0.731	0.667	0.718	0.640	0.601	0.739	**0.746**
	Err	0.355	**0.314**	0.359	0.441	0.419	0.456	0.320	**0.293**	0.306	0.424	0.431	0.429	0.365	**0.319**	0.373	0.445	0.421	0.468
30	BS	0.011	**0.010**	0.173	0.249	0.254	0.259	0.014	**0.013**	0.203	0.262	0.278	0.274	0.019	**0.014**	0.177	0.274	0.289	0.273
	sen	0.741	**0.755**	0.639	0.718	0.701	0.687	0.773	**0.784**	0.686	0.732	0.735	0.739	0.684	0.714	0.650	0.646	0.689	**0.773**

Table 3. Classification error rate, sensitivity and Brier score produced by Random Forest, k Nearest Neighbors and Support Vector Machine classifiers on Srbct dataset when the data is partioned into (30%) training and (70%) testing parts.

		RF						kNN						SVM					
Genes		POS	RPOS	GClust	sigF	Wilc	mRmR	POS	RPOS	GClust	sigF	Wilc	mRmR	POS	RPOS	GClust	sigF	Wilc	mRmR
	Err	0.071	0.061	0.078	**0.054**	0.057	0.109	**0.127**	0.218	0.144	0.163	0.213	0.212	**0.152**	0.300	0.177	0.163	0.431	0.226
5	BS	**0.005**	**0.005**	0.039	0.039	0.062	0.103	**0.014**	0.023	0.099	0.026	0.153	0.154	0.036	**0.020**	0.053	0.036	0.261	0.158
	sen	0.884	0.901	0.903	**0.950**	0.899	0.842	**0.796**	0.639	0.596	0.769	0.729	0.762	**0.829**	0.681	0.659	0.812	0.486	0.731
	Err	0.063	0.038	0.039	**0.036**	0.058	0.499	**0.086**	0.099	0.142	0.119	0.218	0.214	**0.154**	0.283	0.171	0.299	0.414	0.496
10	BS	0.007	**0.005**	0.038	0.036	0.070	0.282	**0.014**	0.016	0.094	**0.014**	0.158	0.155	0.037	**0.017**	0.051	0.037	0.246	0.281
	sen	0.918	0.928	0.911	**0.964**	0.901	0.470	0.893	0.846	0.607	**0.992**	0.761	0.766	**0.833**	0.696	0.643	0.821	0.516	0.429
	Err	0.026	0.030	0.039	**0.011**	0.050	0.396	0.075	0.072	0.173	**0.019**	0.250	0.207	**0.125**	0.160	0.142	0.126	0.379	0.390
15	BS	**0.004**	0.005	0.047	0.031	0.071	0.239	**0.009**	0.011	0.121	0.010	0.167	0.155	0.041	**0.011**	0.035	0.031	0.216	0.243
	sen	0.957	0.975	0.897	**0.988**	0.951	0.597	0.922	**0.941**	0.544	0.917	0.742	0.775	**0.899**	0.844	0.690	0.871	0.590	0.554
	Err	0.025	0.017	0.034	**0.011**	0.052	0.157	0.063	**0.050**	0.137	0.083	0.208	0.210	0.120	**0.077**	0.146	0.123	0.342	0.300
20	BS	0.005	**0.004**	0.050	0.033	0.073	0.143	0.009	**0.008**	0.102	0.010	0.150	0.152	0.032	**0.006**	0.035	0.032	0.200	0.167
	sen	0.956	0.984	0.916	**0.985**	0.944	0.793	0.928	**0.968**	0.655	0.938	0.755	0.762	0.909	**0.928**	0.907	0.912	0.590	0.664
	Err	0.023	0.023	0.040	**0.010**	0.048	0.296	0.059	**0.051**	0.163	0.101	0.223	0.211	0.089	**0.074**	0.149	0.117	0.350	0.424
25	BS	**0.004**	**0.004**	0.054	0.034	0.074	0.195	**0.008**	**0.008**	0.103	0.015	0.157	0.154	0.031	**0.006**	0.037	0.029	0.178	0.229
	sen	0.966	0.986	0.901	**0.989**	0.941	0.633	0.929	**0.972**	0.560	0.937	0.737	0.770	0.959	**0.963**	0.908	0.958	0.582	0.539
	Err	0.019	0.018	0.034	**0.009**	0.049	0.271	0.058	**0.051**	0.160	0.090	0.195	0.205	0.095	**0.064**	0.153	0.107	0.327	0.388
30	BS	**0.004**	**0.004**	0.048	0.035	0.074	0.189	0.008	**0.006**	0.113	0.014	0.149	0.152	0.030	**0.006**	0.036	0.027	0.180	0.214
	sen	0.967	0.987	0.909	**0.993**	0.955	0.697	0.939	0.959	0.563	**0.998**	0.815	0.774	0.959	**0.978**	0.865	0.951	0.652	0.553

Table 4. Classification error rate, sensitivity and Brier score produced by Random Forest, k Nearest Neighbors and Support Vector Machine classifiers on DLBCL dataset when the data is partioned into (70%) training and (30%) testing parts.

		RF						kNN						SVM					
Genes		POS	RPOS	GClust	sigF	Wilc	mRmR	POS	RPOS	GClust	sigG	Wilc	mRmR	POS	RPOS	GClust	sigF	Wilc	mRmR
	Err	0.146	0.118	0.089	0.257	**0.005**	0.318	**0.121**	0.147	0.143	0.291	0.150	0.137	0.153	**0.107**	0.185	0.337	0.252	0.261
5	BS	0.007	**0.005**	0.084	0.174	0.017	0.204	**0.006**	0.007	0.082	0.191	0.150	0.104	0.025	**0.005**	0.058	0.184	0.198	0.201
	sen	0.606	0.694	0.788	0.893	**0.972**	0.127	0.687	0.597	0.867	0.838	**0.872**	0.540	0.761	**0.853**	0.751	0.700	0.040	0.017
	Err	0.076	0.114	0.073	0.252	**0.023**	0.285	0.125	**0.100**	0.162	0.342	0.140	0.133	0.093	**0.073**	0.092	0.258	0.257	0.251
10	BS	**0.004**	**0.004**	0.072	0.172	0.029	0.181	0.005	**0.003**	0.053	0.203	0.102	0.100	0.030	**0.003**	0.040	0.166	0.189	0.196
	sen	0.793	0.732	0.775	0.911	**0.960**	0.176	0.685	0.817	0.859	0.760	**0.923**	0.545	0.745	**0.937**	0.846	0.803	0.090	0.007
	Err	0.074	0.106	0.107	0.191	**0.039**	0.270	0.109	**0.087**	0.154	0.304	0.156	0.136	0.100	**0.047**	0.058	0.176	0.275	0.250
15	BS	**0.003**	0.005	0.076	0.145	0.042	0.172	0.005	**0.004**	0.110	0.198	0.156	0.101	0.034	**0.003**	0.044	0.122	0.184	0.187
	sen	0.783	0.723	0.679	**0.948**	0.910	0.130	0.699	0.853	0.469	0.802	**0.918**	0.551	0.742	**0.974**	0.816	0.886	0.089	0.008
	Err	0.075	0.110	0.079	0.188	**0.039**	0.260	0.103	**0.078**	0.114	0.327	0.169	0.132	0.080	0.063	**0.051**	0.134	0.223	0.248
20	BS	**0.004**	0.005	0.074	0.142	0.038	0.162	0.006	**0.005**	0.087	0.192	0.169	0.101	0.034	**0.003**	0.038	0.099	0.153	0.170
	sen	0.783	0.749	0.774	**0.963**	0.924	0.117	0.831	**0.928**	0.701	0.746	0.905	0.531	0.825	**0.979**	0.863	0.904	0.134	0.016
	Err	0.084	0.119	0.082	0.177	**0.046**	0.276	0.113	**0.089**	0.105	0.329	0.164	0.133	0.092	**0.065**	0.085	0.141	0.246	0.253
25	BS	**0.004**	0.005	0.075	0.140	0.044	0.178	**0.006**	**0.006**	0.077	0.195	0.164	0.100	0.038	**0.003**	0.044	0.103	0.157	0.186
	sen	0.776	0.737	0.748	**0.972**	0.880	0.162	0.758	**0.947**	0.777	0.759	0.884	0.543	0.784	**0.983**	0.863	0.913	0.124	0.010
	Err	0.079	0.120	0.067	0.128	**0.056**	0.268	0.110	**0.098**	0.145	0.196	0.165	0.136	0.086	**0.070**	0.099	0.127	0.245	0.251
30	BS	**0.005**	0.007	0.059	0.116	0.049	0.171	**0.007**	0.009	0.039	0.124	0.165	0.102	0.040	**0.004**	0.026	0.097	0.141	0.187
	sen	0.810	0.729	0.868	**0.983**	0.865	0.155	0.823	**0.936**	0.847	0.832	0.895	0.547	0.826	**0.975**	0.892	0.917	0.089	0.000

Table 5. Classification error rate, sensitivity and Brier score produced by Random Forest, k Nearest Neighbors and Support Vector Machine classifiers on DLBCL dataset when the data is partioned into (30%) training and (70%) testing parts.

		RF						kNN						SVM					
Genes		POS	RPOS	GClust	sigF	Wilc	mRmR	POS	RPOS	GClust	sigF	Wilc	mRmR	POS	RPOS	GClust	sigF	Wilc	mRmR
	Err	0.188	0.147	0.127	0.285	**0.118**	0.242	0.188	**0.147**	0.187	0.295	0.192	0.187	0.218	**0.185**	0.193	0.350	0.262	0.253
5	BS	0.008	**0.007**	0.095	0.192	0.085	0.168	0.008	**0.007**	0.122	0.197	0.192	0.134	0.022	**0.010**	0.082	0.207	0.207	0.192
	sen	0.491	0.600	0.659	**0.883**	0.698	0.398	0.491	0.600	0.437	**0.861**	0.844	0.719	0.504	0.574	0.568	**0.746**	0.056	0.129
	Err	0.183	**0.114**	0.115	0.269	0.121	0.242	0.183	**0.114**	0.179	0.312	0.196	0.186	0.197	**0.153**	0.196	0.313	0.256	0.252
10	BS	0.006	**0.004**	0.088	0.185	0.095	0.167	0.006	**0.004**	0.115	0.196	0.196	0.135	0.016	**0.007**	0.057	0.202	0.200	0.192
	sen	0.395	0.671	0.666	**0.899**	0.658	0.401	0.395	0.671	0.466	0.817	**0.844**	0.723	0.512	0.648	0.659	**0.778**	0.065	0.135
	Err	0.164	**0.110**	0.119	0.235	0.138	0.282	0.164	**0.110**	0.202	0.312	0.176	0.193	0.169	**0.105**	0.113	0.249	0.257	0.256
15	BS	0.005	**0.003**	0.087	0.165	0.098	0.202	0.005	**0.003**	0.128	0.193	0.176	0.137	0.012	**0.004**	0.058	0.175	0.193	0.209
	sen	0.417	0.683	0.632	**0.932**	0.611	0.150	0.417	0.683	0.273	0.814	**0.826**	0.704	0.517	0.749	0.606	**0.833**	0.079	0.025
	Err	0.157	**0.127**	0.158	0.227	0.138	0.250	0.157	**0.127**	0.182	0.328	0.189	0.190	0.154	**0.112**	0.141	0.212	0.251	0.259
20	BS	**0.006**	**0.006**	0.090	0.163	0.100	0.167	**0.006**	**0.006**	0.116	0.199	0.189	0.136	0.012	**0.006**	0.072	0.153	0.187	0.203
	sen	0.499	0.657	0.659	**0.945**	0.642	0.207	0.499	0.657	0.385	0.766	**0.849**	0.683	0.576	0.779	0.524	**0.856**	0.031	0.029
	Err	0.154	**0.130**	0.164	0.223	0.145	0.252	0.154	**0.130**	0.206	0.303	0.197	0.196	0.126	**0.108**	0.137	0.191	0.252	0.252
25	BS	**0.005**	**0.005**	0.094	0.163	0.107	0.177	**0.005**	**0.005**	0.129	0.191	0.197	0.138	0.013	**0.005**	0.074	0.152	0.189	0.194
	sen	0.471	0.644	0.633	**0.951**	0.609	0.253	0.471	0.644	0.327	0.779	**0.852**	0.711	0.613	0.769	0.560	**0.891**	0.087	0.047
	Err	0.153	**0.127**	0.171	0.195	0.167	0.248	0.153	**0.127**	0.178	0.186	0.180	0.192	0.124	**0.109**	0.191	0.172	0.262	0.255
30	BS	**0.004**	**0.004**	0.087	0.143	0.116	0.179	**0.004**	**0.004**	0.116	0.131	0.180	0.136	0.014	**0.005**	0.050	0.135	0.188	0.208
	sen	0.478	0.656	0.690	**0.961**	0.497	0.192	0.478	0.656	0.523	**0.867**	0.792	0.701	0.623	0.773	0.673	**0.915**	0.034	0.013

Table 6. Classification error rate, sensitivity and Brier score produced by Random Forest, k Nearest Neighbors and Support Vector Machine classifiers on nki70 dataset when the data is partioned into (70%) training and (30%) testing parts.

		RF						kNN						SVM					
Genes		POS	RPOS	GClust	sigF	Wilc	mRmR	POS	RPOS	GClust	sigF	Wilc	mRmR	POS	RPOS	GClust	sigF	Wilc	mRmR
	Err	0.313	0.154	0.148	0.321	**0.000**	0.300	0.325	**0.197**	0.199	0.317	0.276	0.333	0.371	0.222	0.247	0.313	0.210	0.327
5	BS	0.006	**0.004**	0.146	0.222	0.005	0.138	0.007	**0.005**	0.154	0.225	0.211	0.160	0.011	**0.005**	0.169	0.205	0.157	0.147
	sen	0.399	0.707	0.643	0.390	**1.000**	0.671	0.465	0.601	0.547	0.420	**0.628**	0.552	0.600	**0.688**	0.431	0.643	0.496	0.545
	Err	0.332	0.154	0.160	0.329	**0.000**	0.292	0.379	**0.187**	0.197	0.343	0.292	0.319	0.389	0.257	0.250	0.326	**0.230**	0.306
10	BS	0.008	**0.005**	0.150	0.216	0.012	0.138	0.010	**0.005**	0.151	0.234	0.221	0.162	0.012	**0.008**	0.173	0.204	0.169	0.160
	sen	0.344	0.676	0.628	0.325	**1.000**	0.650	0.338	**0.636**	0.554	0.349	0.588	0.549	0.569	**0.665**	0.414	0.529	0.465	0.411
	Err	0.148	0.150	0.158	0.320	**0.001**	0.252	**0.149**	0.195	0.530	0.327	0.282	0.301	0.258	**0.224**	0.258	0.304	0.234	0.299
15	BS	**0.006**	**0.006**	0.150	0.206	0.028	0.138	0.006	**0.005**	0.136	0.217	0.211	0.161	0.016	**0.006**	0.177	0.192	0.166	0.168
	sen	0.639	0.641	0.626	0.272	**0.998**	0.643	**0.638**	0.612	0.615	0.388	0.611	0.545	0.652	**0.688**	0.373	0.609	0.464	0.491
	Err	0.152	0.159	0.157	0.312	**0.001**	0.253	**0.195**	0.200	0.204	0.289	0.276	0.291	0.257	0.237	0.251	0.278	**0.225**	0.279
20	BS	**0.006**	**0.006**	0.152	0.200	0.029	0.144	**0.006**	**0.006**	0.155	0.189	0.208	0.164	0.015	**0.007**	0.172	0.176	0.161	0.170
	sen	0.650	0.647	0.624	0.307	**0.998**	0.640	0.604	0.612	0.547	0.420	**0.630**	0.545	0.619	**0.666**	0.396	0.636	0.483	0.457
	Err	0.147	0.156	0.164	0.331	**0.000**	0.242	**0.193**	0.200	0.202	0.298	0.273	0.285	0.271	0.245	0.253	0.290	**0.243**	0.261
25	BS	**0.005**	**0.005**	0.158	0.205	0.030	0.143	**0.005**	**0.005**	0.153	0.201	0.211	0.166	0.011	**0.005**	0.174	0.178	0.166	0.169
	sen	0.653	0.644	0.611	0.268	**1.000**	0.644	0.622	**0.636**	0.543	0.460	0.632	0.537	0.639	**0.676**	0.404	0.640	0.443	0.409
	Err	0.152	0.164	0.161	0.339	**0.004**	0.230	**0.204**	0.209	**0.204**	0.288	0.276	0.269	0.290	0.271	0.264	0.302	**0.235**	0.268
30	BS	**0.005**	**0.005**	0.157	0.208	0.041	0.150	**0.006**	**0.006**	0.154	0.199	0.207	0.163	0.009	**0.006**	0.180	0.185	0.164	0.168
	sen	0.650	0.646	0.620	0.259	**0.988**	0.632	0.630	**0.636**	0.542	0.453	0.621	0.551	0.607	**0.655**	0.344	0.630	0.447	0.394

Table 7. Classification error rate, sensitivity and Brier score produced by Random Forest, k Nearest Neighbors and Support Vector Machine classifiers on nki70 dataset when the data is partioned into (30%) training and (70%) testing parts.

		RF						kNN						SVM					
Genes		POS	RPOS	GClust	sigF	Wilc	mRmR	POS	RPOS	GClust	sigF	Wilc	mRmR	POS	RPOS	GClust	sigF	Wilc	mRmR
	Err	0.360	0.182	0.182	0.341	**0.000**	0.379	0.374	**0.224**	0.256	0.332	0.287	0.322	0.393	0.294	0.258	0.347	**0.240**	0.348
5	BS	0.007	**0.005**	0.156	0.225	0.013	0.245	0.008	**0.005**	0.138	0.227	0.212	0.173	0.009	**0.005**	0.180	0.212	0.173	0.236
	sen	0.315	0.640	0.605	0.341	**1.000**	0.265	0.274	0.527	0.609	0.415	**0.598**	0.533	0.486	**0.588**	0.395	0.583	0.441	0.126
	Err	0.380	0.190	0.194	0.330	**0.005**	0.177	0.385	**0.227**	0.236	0.344	0.276	0.302	0.430	0.313	0.278	0.347	**0.267**	0.286
10	BS	0.007	**0.005**	0.162	0.216	0.031	0.154	0.009	**0.006**	0.177	0.232	0.207	0.173	0.012	**0.006**	0.191	0.215	0.187	0.200
	sen	0.257	0.608	0.582	0.312	**0.988**	0.621	0.263	0.524	0.449	0.314	**0.604**	0.542	0.459	**0.570**	0.336	0.517	0.386	0.306
	Err	0.360	0.204	0.192	0.323	**0.037**	0.194	0.374	**0.228**	0.255	0.332	0.280	0.291	0.410	0.290	0.282	0.336	**0.279**	0.288
15	BS	0.007	**0.006**	0.162	0.207	0.058	0.161	0.008	**0.006**	0.137	0.218	0.206	0.172	0.015	**0.006**	0.190	0.212	0.190	0.183
	sen	0.229	0.581	0.588	0.304	**0.902**	0.596	0.278	0.534	0.615	0.400	**0.582**	0.545	0.454	**0.581**	0.340	0.535	0.303	0.379
	Err	0.360	0.203	0.193	0.319	**0.029**	0.201	0.373	**0.225**	0.235	0.311	0.275	0.282	0.397	0.293	0.299	0.310	**0.285**	0.293
20	BS	0.007	**0.005**	0.164	0.201	0.057	0.164	0.008	**0.006**	0.176	0.211	0.204	0.174	0.012	**0.006**	0.190	0.201	0.188	0.195
	sen	0.240	0.563	0.574	0.312	**0.921**	0.555	0.266	0.520	0.438	0.397	**0.609**	0.529	0.458	0.544	0.302	**0.556**	0.289	0.272
	Err	0.359	0.211	0.209	0.326	**0.034**	0.202	0.367	**0.211**	0.237	0.321	0.279	0.227	0.408	0.315	0.298	0.312	0.289	**0.280**
25	BS	0.007	**0.006**	0.171	0.205	0.062	0.168	0.007	**0.005**	0.178	0.215	0.207	0.174	0.012	**0.006**	0.197	0.200	0.191	0.187
	sen	0.237	0.537	0.535	0.301	**0.906**	0.556	0.268	0.551	0.438	0.396	**0.589**	0.530	0.464	0.532	0.237	**0.548**	0.259	0.292
	Err	0.362	0.218	0.202	0.339	**0.051**	0.216	0.363	**0.210**	0.233	0.319	0.286	0.226	0.399	0.301	0.296	0.322	**0.293**	0.306
30	BS	**0.005**	**0.005**	0.172	0.208	0.074	0.173	0.007	**0.005**	0.175	0.213	0.207	0.175	0.014	**0.004**	0.203	0.204	0.194	0.198
	sen	0.210	0.526	0.554	0.264	**0.864**	0.533	0.298	0.537	0.455	0.383	**0.591**	0.527	0.462	**0.565**	0.257	0.540	0.260	0.240

Table 8. Classification error rate, sensitivity and Brier score produced by Random Forest, k Nearest Neighbors and Support Vector Machine classifiers on Prostate dataset when the data is partioned into (70%) training and (30%) testing parts.

		RF						kNN						SVM					
Genes		POS	RPOS	GClust	sigF	Wilc	mRmR	POS	RPOS	GClust	sigF	Wilc	mRmR	POS	RPOS	GClust	sigF	Wilc	mRmR
	Err	**0.006**	0.007	0.010	0.197	0.130	0.167	0.005	**0.004**	0.020	0.218	0.011	0.012	**0.016**	0.017	0.019	0.193	0.125	0.170
5	BS	**0.000**	**0.000**	0.009	0.136	0.095	0.134	**0.000**	**0.000**	0.015	0.146	0.011	0.012	0.005	**0.000**	0.007	0.146	0.093	0.136
	sen	**0.972**	0.962	0.953	0.910	0.430	0.133	**0.979**	0.976	0.964	0.968	0.940	0.929	0.919	**0.950**	0.901	0.903	0.399	0.001
	Err	0.006	**0.005**	0.008	0.205	0.121	0.163	0.005	**0.004**	0.014	0.161	0.011	0.013	0.014	**0.010**	0.012	0.119	0.126	0.169
10	BS	**0.000**	**0.000**	0.007	0.133	0.083	0.126	**0.000**	**0.000**	0.011	0.120	0.011	0.012	0.005	**0.000**	0.007	0.087	0.079	0.122
	sen	0.966	**0.971**	0.951	0.927	0.402	0.153	0.972	**0.973**	0.954	0.968	0.931	0.927	0.952	0.939	0.921	**0.954**	0.493	0.034
	Err	**0.006**	0.007	0.009	0.186	0.116	0.143	0.006	**0.005**	0.011	0.200	0.011	0.012	0.020	**0.013**	0.014	0.154	0.107	0.135
15	BS	**0.000**	**0.000**	0.008	0.126	0.085	0.112	**0.000**	**0.000**	0.010	0.148	0.012	0.012	0.003	**0.000**	0.010	0.099	0.069	0.104
	sen	**0.965**	0.960	0.959	0.953	0.376	0.260	0.967	**0.970**	0.943	0.940	0.934	0.931	**0.974**	0.932	0.951	0.942	0.524	0.259
	Err	**0.006**	0.007	0.007	0.185	0.117	0.133	**0.006**	**0.006**	0.011	0.199	0.013	0.012	0.024	**0.012**	0.014	0.118	0.095	0.131
20	BS	**0.000**	**0.000**	0.007	0.127	0.084	0.106	**0.000**	**0.000**	0.010	0.150	0.013	0.012	0.004	**0.000**	0.007	0.079	0.063	0.099
	sen	**0.964**	0.962	0.961	0.940	0.345	0.302	0.967	**0.968**	0.943	0.921	0.923	0.929	**0.975**	0.937	0.941	0.955	0.523	0.284
	Err	0.006	**0.005**	0.008	0.191	0.118	0.127	**0.005**	**0.005**	0.012	0.214	0.012	0.012	0.016	**0.010**	0.013	0.100	0.094	0.117
25	BS	**0.000**	**0.000**	0.008	0.130	0.081	0.101	**0.000**	**0.000**	0.010	0.152	0.012	0.012	0.003	**0.000**	0.008	0.078	0.016	0.091
	sen	0.967	**0.971**	0.968	0.937	0.302	0.342	0.971	**0.973**	0.955	0.904	0.926	0.928	**0.972**	0.948	0.961	0.961	0.507	0.358
	Err	**0.006**	**0.006**	0.008	0.197	0.070	0.134	0.006	**0.005**	0.011	0.210	0.013	0.013	0.014	**0.010**	0.017	0.109	0.071	0.117
30	BS	**0.000**	**0.000**	0.008	0.134	0.057	0.104	**0.000**	**0.000**	0.009	0.137	0.012	0.012	0.003	**0.000**	0.008	0.090	0.047	0.090
	sen	0.964	0.961	**1.000**	0.938	0.626	0.297	0.965	**0.968**	0.960	0.916	0.927	0.928	**0.966**	0.939	0.911	0.943	0.667	0.346

Table 9. Classification error rate, sensitivity and Brier score produced by Random Forest, k Nearest Neighbors and Support Vector Machine classifiers on Prostate dataset when the data is partioned into (30%) training and (70%) testing parts.

		RF						kNN						SVM					
Genes		POS	RPOS	GClust	sigF	Wilc	mRmR	POS	RPOS	GClust	sigF	Wilc	mRmR	POS	RPOS	GClust	sigF	Wilc	mRmR
	Err	**0.006**	0.009	0.011	0.234	0.070	0.167	0.011	**0.007**	0.030	0.212	0.014	0.013	**0.038**	0.075	0.084	0.233	0.062	0.171
5	BS	**0.000**	**0.000**	0.011	0.155	0.052	0.132	**0.000**	**0.000**	0.023	0.162	0.014	0.013	0.308	**0.001**	0.013	0.166	0.051	0.136
	sen	0.967	**0.969**	0.929	0.893	0.769	0.158	0.937	**0.977**	0.916	0.954	0.921	0.923	0.979	**1.000**	0.998	0.852	0.816	0.029
	Err	**0.006**	0.008	0.009	0.213	0.066	0.141	**0.005**	0.006	0.016	0.227	0.013	0.013	**0.039**	0.072	0.044	0.170	0.046	0.145
10	BS	**0.000**	**0.000**	0.009	0.154	0.052	0.110	**0.000**	**0.000**	0.014	0.169	0.014	0.013	0.316	**0.001**	0.010	0.127	0.042	0.110
	sen	**0.963**	**0.963**	0.954	0.924	0.750	0.278	0.971	**0.984**	0.900	0.919	0.925	0.925	0.987	**0.988**	0.956	0.922	0.849	0.206
	Err	**0.006**	0.007	0.010	0.220	0.070	0.162	**0.005**	**0.005**	0.021	0.262	0.013	0.013	**0.034**	0.043	0.057	0.183	0.046	0.163
15	BS	**0.000**	**0.000**	0.009	0.160	0.054	0.125	**0.000**	**0.000**	0.017	0.190	0.014	0.013	0.074	**0.000**	0.013	0.138	0.039	0.119
	sen	0.963	**0.965**	0.919	0.932	0.714	0.133	0.972	**0.974**	0.941	0.868	0.926	0.923	0.984	0.985	**0.997**	0.921	0.827	0.070
	Err	**0.007**	**0.007**	0.010	0.216	0.057	0.140	**0.005**	**0.005**	0.019	0.255	0.013	0.013	**0.030**	0.042	0.067	0.168	0.038	0.128
20	BS	**0.000**	**0.000**	0.009	0.160	0.049	0.109	**0.000**	**0.000**	0.016	0.185	0.013	0.012	0.102	**0.000**	0.012	0.130	0.033	0.098
	sen	0.964	**0.967**	0.938	0.926	0.766	0.272	0.970	**0.973**	0.923	0.872	0.923	0.925	**0.985**	**0.985**	0.970	0.943	0.867	0.296
	Err	**0.007**	**0.007**	0.008	0.216	0.051	0.134	**0.005**	**0.005**	0.017	0.252	0.013	0.013	**0.030**	0.042	0.061	0.179	0.035	0.114
25	BS	**0.000**	**0.000**	0.010	0.163	0.044	0.104	**0.000**	**0.000**	0.015	0.183	0.013	0.013	0.102	**0.000**	0.010	0.135	0.032	0.088
	sen	0.964	**0.967**	0.954	0.931	0.801	0.288	0.970	**0.973**	0.965	0.872	0.926	0.923	**0.985**	**0.985**	0.923	0.938	0.877	0.348
	Err	**0.007**	**0.007**	0.008	0.230	0.048	0.149	**0.005**	**0.005**	0.014	0.247	0.013	0.013	**0.014**	0.015	0.031	0.186	0.033	0.127
30	BS	**0.000**	**0.000**	0.009	0.166	0.044	0.110	**0.000**	**0.000**	0.012	0.186	0.013	0.013	0.084	**0.000**	0.010	0.144	0.030	0.095
	sen	0.963	**0.966**	0.933	0.921	0.803	0.198	**0.973**	0.972	0.921	0.889	0.927	0.926	0.955	**0.961**	0.901	0.929	0.877	0.278

Table 10. Classification error rate, sensitivity and Brier score produced by Random Forest, k Nearest Neighbors and Support Vector Machine classifiers on Lungcancer dataset when the data is partioned into (70%) training and (30%) testing parts.

		RF						kNN						SVM					
Genes		POS	RPOS	GClust	sigF	Wilc	mRmR	POS	RPOS	GClust	sigF	Wilc	mRmR	POS	RPOS	GClust	sigF	Wilc	mRmR
	Err	0.008	**0.007**	0.009	0.100	0.076	0.082	0.005	**0.001**	0.034	0.094	0.094	0.042	0.039	**0.015**	0.050	0.251	0.090	0.093
5	BS	**0.000**	**0.000**	0.007	0.086	0.073	0.086	**0.000**	**0.000**	0.024	0.099	0.086	0.024	0.001	**0.000**	0.020	0.085	0.069	0.084
	sen	0.942	**0.923**	0.891	0.111	0.274	0.173	0.935	**0.981**	0.681	0.000	0.000	0.588	0.611	**0.841**	0.552	0.214	0.014	0.000
	Err	0.013	**0.007**	0.010	0.088	0.088	0.085	**0.003**	0.009	0.056	0.090	0.093	0.043	0.016	**0.010**	0.052	0.214	0.093	0.094
10	BS	**0.000**	**0.000**	0.010	0.076	0.073	0.070	**0.000**	**0.000**	0.038	0.094	0.086	0.025	0.002	**0.000**	0.018	0.084	0.082	0.069
	sen	0.926	**0.928**	0.834	0.130	0.223	0.161	**0.970**	0.938	0.455	0.019	0.000	0.585	0.896	**0.905**	0.558	0.258	0.000	0.000
	Err	**0.013**	**0.013**	0.017	0.080	0.089	0.090	**0.001**	0.010	0.065	0.093	0.094	0.044	0.022	**0.013**	0.027	0.194	0.094	0.093
15	BS	**0.000**	**0.000**	0.010	0.071	0.073	0.076	**0.000**	**0.000**	0.041	0.098	0.086	0.025	0.002	**0.000**	0.014	0.085	0.079	0.071
	sen	**0.906**	0.853	0.834	0.181	0.189	0.071	**0.983**	0.893	0.355	0.005	0.000	0.574	0.845	**0.847**	0.833	0.183	0.000	0.000
	Err	**0.010**	0.014	0.014	0.079	0.088	0.086	**0.006**	0.007	0.052	0.093	0.094	0.042	0.018	**0.012**	0.033	0.155	0.095	0.093
20	BS	**0.000**	**0.000**	0.009	0.070	0.074	0.071	**0.000**	**0.000**	0.032	0.097	0.087	0.024	0.003	**0.000**	0.015	0.084	0.077	0.078
	sen	**0.941**	0.861	0.966	0.203	0.148	0.117	**0.937**	0.930	0.488	0.005	0.000	0.589	**0.893**	0.880	0.814	0.174	0.000	0.000
	Err	0.013	0.016	0.014	0.081	0.098	0.084	0.009	**0.006**	0.038	0.092	0.104	0.044	0.016	**0.007**	0.027	0.129	0.104	0.094
25	BS	**0.000**	**0.000**	0.009	0.066	0.078	0.067	**0.000**	**0.000**	0.025	0.096	0.096	0.025	0.002	**0.000**	0.013	0.081	0.082	0.072
	sen	**0.904**	0.813	0.873	0.212	0.185	0.161	0.891	**0.926**	0.641	0.008	0.000	0.578	0.895	**0.911**	0.832	0.162	0.000	0.000
	Err	0.009	0.013	0.012	0.055	0.087	0.082	**0.002**	0.005	0.050	0.088	0.094	0.044	0.012	**0.007**	0.014	0.058	0.094	0.094
30	BS	**0.000**	**0.000**	0.008	0.036	0.072	0.069	**0.000**	**0.000**	0.030	0.077	0.087	0.025	0.004	**0.000**	0.009	0.045	0.073	0.074
	sen	**0.959**	0.869	0.877	0.511	0.167	0.145	**0.987**	0.953	0.527	**0.027**	0.000	0.567	**0.960**	0.930	0.908	0.422	0.000	0.000

Table 11. Classification error rate, sensitivity and Brier score produced by Random Forest, k Nearest Neighbors and Support Vector Machine classifiers on Lungcancer dataset when the data is partioned into (30%) training and (70%) testing parts.

		RF						kNN						SVM					
Genes		POS	RPOS	GClust	sigF	Wilc	mRmR	POS	RPOS	GClust	sigF	Wilc	mRmR	POS	RPOS	GClust	sigF	Wilc	mRmR
	Err	0.022	**0.012**	0.015	0.103	0.096	0.102	0.040	**0.019**	0.599	0.099	0.094	0.071	0.065	**0.020**	0.070	0.282	0.095	0.093
5	BS	**0.000**	**0.000**	0.011	0.093	0.078	0.093	**0.001**	**0.001**	0.046	0.106	0.088	0.048	0.003	**0.000**	0.028	0.087	0.083	0.088
	sen	0.843	**0.879**	0.872	0.052	0.127	0.020	0.668	**0.826**	0.320	0.000	0.000	0.254	0.371	**0.793**	0.771	0.198	0.000	0.000
	Err	**0.008**	0.010	0.015	0.095	0.095	0.099	**0.012**	0.013	0.635	0.101	0.093	0.072	0.038	**0.011**	0.069	0.192	0.093	0.093
10	BS	**0.000**	**0.000**	0.014	0.086	0.078	0.088	**0.001**	**0.001**	0.056	0.104	0.086	0.048	0.014	**0.000**	0.028	0.092	0.085	0.087
	sen	**0.964**	0.900	0.954	0.088	0.069	0.033	**0.896**	0.872	0.267	0.005	0.000	0.253	0.663	**0.882**	0.853	0.168	0.000	0.001
	Err	**0.004**	0.010	0.019	0.092	0.103	0.100	**0.022**	0.028	0.773	0.096	0.099	0.071	0.039	**0.020**	0.060	0.143	0.099	0.093
15	BS	**0.000**	**0.000**	0.016	0.082	0.081	0.088	**0.002**	**0.002**	0.061	0.106	0.091	0.048	0.012	**0.000**	0.023	0.091	0.090	0.086
	sen	**0.983**	0.895	0.876	0.093	0.006	0.029	**0.811**	0.754	0.117	0.002	0.000	0.259	0.655	**0.812**	0.762	0.099	0.000	0.001
	Err	**0.012**	0.014	0.015	0.091	0.086	0.097	**0.042**	0.043	0.638	0.096	0.084	0.072	0.036	**0.024**	0.063	0.126	0.084	0.093
20	BS	**0.000**	**0.000**	0.015	0.081	0.069	0.085	**0.002**	**0.002**	0.057	0.106	0.079	0.048	0.073	**0.001**	0.023	0.090	0.076	0.083
	sen	**0.927**	0.876	0.851	0.100	0.006	0.049	**0.599**	0.568	0.257	0.002	0.000	0.253	0.673	**0.777**	0.747	0.098	0.000	0.002
	Err	**0.008**	0.014	0.012	0.090	0.089	0.099	0.037	**0.035**	0.600	0.097	0.092	0.072	0.030	**0.019**	0.052	0.112	0.092	0.094
25	BS	**0.000**	**0.000**	0.014	0.079	0.074	0.088	**0.002**	**0.002**	0.048	0.106	0.085	0.049	0.072	**0.001**	0.018	0.089	0.081	0.086
	sen	**0.944**	0.866	0.870	0.100	0.040	0.029	0.631	**0.647**	0.320	0.002	0.000	0.248	0.731	**0.831**	0.768	0.078	0.000	0.000
	Err	**0.011**	0.013	0.019	0.088	0.088	0.098	0.040	**0.039**	0.604	0.097	0.075	0.072	0.034	**0.023**	0.048	0.075	0.093	0.093
30	BS	**0.000**	**0.000**	0.013	0.055	0.074	0.085	**0.002**	**0.002**	0.052	0.092	0.069	0.048	0.085	**0.001**	0.022	0.066	0.080	0.083
	sen	**0.924**	0.869	0.878	0.141	0.063	0.046	0.611	**0.622**	0.327	0.002	0.000	0.251	0.695	**0.786**	0.777	0.264	0.000	0.005

Table 12. Classification error rate, sensitivity and Brier score produced by Random Forest, k Nearest Neighbors and Support Vector Machine classifiers on Colon dataset when the data is partioned into (70%) training and (30%) testing parts.

		RF						kNN						SVM					
Genes		POS	RPOS	GClust	sigF	Wilc	mRmR	POS	RPOS	GClust	sigF	Wilc	mRmR	POS	RPOS	GClust	sigF	Wilc	mRmR
	Err	0.386	0.370	0.284	0.197	0.334	**0.196**	0.414	0.383	0.300	0.218	**0.209**	0.211	0.416	0.389	0.288	**0.193**	0.364	0.207
5	BS	0.018	**0.016**	0.344	0.136	0.213	0.147	0.022	**0.020**	0.181	0.146	0.167	0.167	0.023	**0.017**	0.346	0.146	0.264	0.155
	sen	0.751	0.759	0.709	**0.910**	0.817	0.891	0.687	0.743	0.285	**0.968**	0.936	0.954	0.644	0.685	0.678	**0.903**	0.811	0.874
	Err	0.392	0.341	0.281	**0.205**	0.293	0.211	0.425	0.375	0.290	**0.161**	0.253	0.316	0.439	0.373	0.295	**0.119**	0.346	0.232
10	BS	0.021	**0.018**	0.345	0.133	0.194	0.173	0.025	**0.022**	0.187	0.120	0.184	0.175	0.030	**0.020**	0.335	0.087	0.255	0.198
	sen	0.771	0.809	0.697	**0.927**	0.873	0.923	0.729	0.768	0.265	**0.968**	0.922	0.923	0.626	0.705	0.635	**0.954**	0.837	0.709
	Err	0.389	0.357	0.247	**0.186**	0.303	0.211	0.438	0.386	0.292	**0.200**	0.287	0.316	0.467	0.366	0.277	**0.154**	0.338	0.245
15	BS	0.017	**0.017**	0.334	0.126	0.194	0.163	**0.024**	0.027	0.172	0.148	0.198	0.175	0.024	**0.020**	0.351	0.099	0.254	0.175
	sen	0.825	0.851	0.771	**0.953**	0.862	0.923	0.693	0.807	0.249	**0.940**	0.850	0.923	0.625	0.765	0.677	**0.942**	0.844	0.872
	Err	0.393	0.342	0.264	**0.185**	0.293	0.203	0.427	0.401	0.342	**0.199**	0.366	0.316	0.439	0.393	0.248	**0.118**	0.329	0.158
20	BS	**0.017**	**0.017**	0.246	0.127	0.187	0.152	0.024	**0.032**	0.167	0.150	0.210	0.175	0.026	**0.020**	0.286	0.079	0.240	0.146
	sen	0.838	0.875	0.721	**0.940**	0.860	0.923	0.747	0.783	0.442	0.921	0.557	**0.923**	0.693	0.735	0.760	**0.955**	0.855	0.923
	Err	0.378	0.325	0.247	**0.191**	0.290	0.199	0.413	0.367	0.382	**0.214**	0.469	0.316	0.442	0.337	0.263	**0.100**	0.336	0.211
25	BS	0.014	**0.013**	0.334	0.130	0.188	0.156	**0.020**	0.027	0.167	0.152	0.222	0.175	0.027	**0.020**	0.365	0.078	0.246	0.146
	sen	0.834	0.885	0.771	**0.937**	0.872	0.923	0.726	0.786	0.350	0.904	0.325	**0.923**	0.664	0.776	0.708	**0.961**	0.873	0.923
	Err	0.356	0.326	0.246	0.197	0.286	**0.166**	0.405	0.371	0.324	**0.210**	0.494	0.221	0.408	0.366	0.224	**0.109**	0.331	0.160
30	BS	0.015	**0.014**	0.309	0.134	0.184	0.136	**0.019**	0.028	0.156	0.137	0.224	0.176	0.020	**0.019**	0.156	0.090	0.247	0.139
	sen	0.870	0.897	0.789	**0.938**	0.885	0.897	0.726	0.812	0.488	0.916	0.276	**0.938**	0.722	0.759	0.488	**0.943**	0.888	0.886

Table 13. Classification error rate, sensitivity and Brier score produced by Random Forest, k Nearest Neighbors and Support Vector Machine classifiers on Colon dataset when the data is partioned into (30%) training and (70%) testing parts.

		RF						kNN						SVM					
Genes		POS	RPOS	GClust	sigF	Wilc	mRmR	POS	RPOS	GClust	sigF	Wilc	mRmR	POS	RPOS	GClust	sigF	Wilc	mRmR
	Err	0.423	0.425	0.381	**0.234**	0.375	0.283	0.440	0.431	0.375	**0.212**	0.294	0.328	0.441	0.440	0.407	**0.233**	0.412	0.298
5	BS	**0.016**	0.019	0.331	0.155	0.255	0.199	**0.028**	**0.028**	0.180	0.162	0.210	0.218	0.027	**0.024**	0.290	0.166	0.294	0.213
	sen	0.709	0.695	0.703	**0.893**	0.751	0.806	0.697	0.702	0.475	**0.954**	0.881	0.803	0.651	0.653	0.637	**0.852**	0.777	0.782
	Err	0.434	0.428	0.388	**0.213**	0.378	0.263	0.446	0.439	0.353	**0.227**	0.433	0.311	0.452	0.442	0.426	**0.170**	0.397	0.265
10	BS	**0.017**	**0.017**	0.300	0.154	0.244	0.186	0.027	**0.026**	0.177	0.169	0.228	0.211	**0.023**	**0.023**	0.285	0.127	0.281	0.195
	sen	0.701	0.707	0.689	**0.924**	0.765	0.867	0.672	0.704	0.419	**0.919**	0.535	0.842	0.642	0.672	0.560	**0.922**	0.810	0.823
	Err	0.427	0.419	0.361	**0.220**	0.371	0.268	0.459	0.433	0.346	**0.262**	0.592	0.306	0.442	0.442	0.399	**0.183**	0.391	0.270
15	BS	0.019	**0.018**	0.277	0.160	0.238	0.187	**0.037**	**0.037**	0.165	0.190	0.239	0.209	0.024	**0.020**	0.301	0.138	0.277	0.195
	sen	0.744	0.751	0.729	**0.932**	0.793	0.854	0.659	0.721	0.461	**0.868**	0.089	0.837	0.686	0.673	0.631	**0.921**	0.842	0.809
	Err	0.421	0.413	0.373	**0.216**	0.357	0.256	0.456	0.433	0.337	**0.255**	0.627	0.314	0.445	0.430	0.362	**0.168**	0.387	0.271
20	BS	**0.015**	**0.015**	0.286	0.160	0.231	0.181	**0.035**	**0.035**	0.153	0.185	0.245	0.215	0.017	**0.016**	0.317	0.130	0.270	0.195
	sen	0.774	0.790	0.697	**0.926**	0.812	0.849	0.664	0.718	0.566	**0.872**	0.035	0.826	0.680	0.711	0.723	**0.943**	0.855	0.792
	Err	0.424	0.410	0.369	**0.216**	0.365	0.266	0.453	0.425	0.345	**0.252**	0.629	0.336	0.449	0.428	0.386	**0.179**	0.383	0.275
25	BS	0.017	**0.015**	0.265	0.163	0.231	0.187	**0.037**	0.039	0.158	0.183	0.246	0.217	0.019	**0.018**	0.293	0.135	0.268	0.193
	sen	0.784	0.811	0.726	**0.931**	0.797	0.844	0.679	0.750	0.472	**0.872**	0.027	0.770	0.688	0.724	0.647	**0.938**	0.864	0.790
	Err	0.414	0.401	0.356	**0.230**	0.373	0.274	0.449	0.422	0.324	**0.247**	0.632	0.302	0.442	0.418	0.344	**0.186**	0.393	0.265
30	BS	0.020	**0.018**	0.249	0.166	0.238	0.187	**0.040**	0.042	0.147	0.186	0.246	0.210	**0.023**	**0.023**	0.266	0.144	0.275	0.189
	sen	0.781	0.794	0.753	**0.921**	0.799	0.855	0.676	0.736	0.568	**0.889**	0.016	0.824	0.681	0.709	0.751	**0.929**	0.861	0.815

Table 14. Classification error rate, sensitivity and Brier score produced by Random Forest, k Nearest Neighbors and Support Vector Machine classifiers on Leukemia dataset when the data is partioned into (70%) training and (30%) testing parts.

		RF						kNN						SVM					
Genes		POS	RPOS	GClust	sigF	Wilc	mRmR	POS	RPOS	GClust	sigF	Wilc	mRmR	POS	RPOS	GClust	sigF	Wilc	mRmR
5	Err	**0.003**	0.032	0.040	0.171	0.050	0.241	**0.079**	0.094	0.089	0.224	0.130	0.286	0.067	**0.055**	0.076	0.166	0.070	0.257
	BS	**0.001**	0.002	0.004	0.115	0.041	0.035	0.004	**0.003**	0.033	0.180	0.100	0.094	0.020	**0.003**	0.015	0.139	0.056	0.028
	sen	**1.000**	0.966	0.953	0.761	0.968	0.842	**0.990**	0.860	0.973	0.547	0.935	0.853	**0.964**	0.958	0.937	0.804	0.955	0.888
10	Err	**0.002**	0.036	0.029	0.173	0.021	0.215	0.093	**0.069**	0.077	0.237	0.136	0.219	0.075	**0.046**	0.133	0.159	**0.046**	0.241
	BS	**0.001**	0.002	0.049	0.108	0.022	0.006	**0.004**	**0.004**	0.040	0.175	0.098	0.101	0.049	**0.002**	0.098	0.126	0.038	0.009
	sen	**1.000**	0.958	0.998	0.765	0.991	0.834	0.929	**0.997**	0.969	0.529	0.873	0.905	0.933	**0.987**	0.935	0.817	0.972	0.853
15	Err	**0.006**	0.043	0.025	0.156	0.040	0.223	0.093	**0.063**	0.069	0.219	0.277	0.201	0.070	**0.043**	0.137	0.166	0.061	0.219
	BS	**0.002**	0.003	0.044	0.099	0.040	0.007	0.006	**0.005**	0.032	0.182	0.162	0.098	0.070	**0.003**	0.088	0.126	0.051	0.006
	sen	**1.000**	0.967	0.999	0.818	0.975	0.895	**1.000**	0.995	0.986	0.509	0.206	0.923	0.969	**0.993**	0.908	0.773	**0.953**	0.871
20	Err	**0.006**	0.035	0.025	0.166	0.149	0.216	0.107	**0.050**	0.222	0.232	0.130	0.243	0.110	**0.034**	0.138	0.187	0.150	0.211
	BS	**0.001**	0.002	0.047	0.106	0.042	0.010	**0.004**	**0.004**	0.142	0.196	0.188	0.095	0.019	**0.002**	0.100	0.141	0.049	0.019
	sen	**1.000**	0.977	0.999	0.802	0.981	0.885	0.990	**0.992**	0.505	0.523	0.178	0.921	0.958	**0.987**	0.923	0.743	0.948	0.933
25	Err	**0.006**	0.025	0.031	0.113	0.007	0.213	0.089	**0.065**	0.113	0.172	0.302	0.313	0.114	**0.035**	0.126	0.136	**0.028**	0.201
	BS	**0.001**	0.005	0.012	0.091	0.015	0.009	0.005	**0.006**	0.063	0.146	0.180	0.094	0.039	**0.006**	0.016	0.100	0.024	0.008
	sen	0.996	0.974	0.979	0.923	**0.998**	0.884	0.980	**0.998**	0.768	0.630	0.071	0.925	0.950	0.969	0.988	0.897	**0.990**	0.911
30	Err	**0.002**	0.040	0.030	0.110	0.006	0.192	0.070	0.081	0.109	0.134	0.124	0.193	0.073	0.055	0.086	0.129	**0.028**	0.191
	BS	**0.001**	0.002	0.013	0.088	0.017	0.012	0.003	0.004	0.054	0.109	0.007	0.094	0.021	**0.002**	0.015	0.090	0.023	0.012
	sen	**1.000**	0.978	0.929	0.923	**1.000**	0.888	0.978	**0.998**	0.759	0.743	0.921	0.928	**0.986**	0.961	0.984	0.900	0.982	0.901

Table 15. Classification error rate, sensitivity and Brier score produced by Random Forest, k Nearest Neighbors and Support Vector Machine classifiers on Leukemia dataset when the data is partioned into (30%) training and (70%) testing parts.

		RF						kNN						SVM					
Genes		POS	RPOS	GClust	sigF	Wilc	mRmR	POS	RPOS	GClust	sigF	Wilc	mRmR	POS	RPOS	GClust	sigF	Wilc	mRmR
	Err	**0.107**	0.119	0.113	0.192	0.127	0.298	**0.125**	0.152	0.183	0.264	0.162	0.168	0.136	**0.125**	0.135	0.215	0.129	0.203
5	BS	**0.003**	0.005	0.014	0.120	0.089	0.062	0.011	**0.010**	0.095	0.206	0.121	0.123	0.118	**0.006**	0.028	0.161	0.097	0.064
	sen	0.851	0.783	0.791	0.642	**0.860**	0.833	0.722	0.694	0.536	0.350	**0.744**	0.735	0.860	0.827	0.868	0.726	**0.869**	0.763
	Err	**0.114**	0.116	0.122	0.205	0.120	0.255	0.179	**0.115**	0.191	0.286	0.298	0.165	0.120	**0.117**	0.151	0.192	0.184	0.194
10	BS	**0.005**	**0.005**	0.023	0.122	0.087	0.039	0.016	**0.007**	0.097	0.214	0.193	0.122	0.102	**0.006**	0.042	0.146	0.097	0.044
	sen	0.817	0.814	0.788	0.606	**0.889**	0.869	0.583	**0.847**	0.516	0.290	0.355	0.755	0.829	0.843	0.784	0.682	**0.845**	0.796
	Err	0.115	0.121	**0.113**	0.199	**0.113**	0.222	0.127	**0.123**	0.163	0.310	0.323	0.167	0.136	**0.105**	0.135	0.204	0.152	0.168
15	BS	0.006	**0.005**	0.022	0.116	0.080	0.040	0.012	**0.007**	0.081	0.237	0.202	0.122	0.098	**0.005**	0.034	0.146	0.081	0.070
	sen	0.809	0.822	0.797	0.596	**0.935**	0.891	0.725	**0.812**	0.534	0.166	0.115	0.755	0.807	0.873	0.755	0.652	**0.913**	0.809
	Err	0.102	0.128	0.111	0.201	**0.099**	0.212	**0.104**	0.144	0.198	0.294	0.322	0.172	0.130	**0.127**	0.179	0.210	0.139	0.154
20	BS	**0.005**	**0.005**	0.085	0.118	0.074	0.050	0.011	**0.009**	0.101	0.230	0.228	0.125	0.098	**0.007**	0.114	0.149	0.081	0.052
	sen	0.862	0.820	0.824	0.616	**0.904**	0.830	**0.803**	0.778	0.502	0.245	0.000	0.733	0.830	0.858	0.762	0.642	**0.910**	0.857
	Err	0.109	**0.094**	0.125	0.170	0.095	0.200	**0.102**	0.107	0.184	0.265	0.324	0.163	0.137	**0.109**	0.143	0.156	0.112	0.148
25	BS	0.005	**0.004**	0.033	0.107	0.079	0.041	0.008	**0.006**	0.105	0.196	0.236	0.121	0.099	**0.006**	0.034	0.113	0.080	0.068
	sen	0.859	0.889	0.794	0.675	**0.923**	0.830	0.826	**0.869**	0.528	0.293	0.000	0.750	0.826	0.894	0.987	0.776	**0.899**	0.886
	Err	0.104	**0.075**	0.117	0.159	0.106	0.177	**0.097**	0.111	0.169	0.231	0.318	0.164	0.116	**0.097**	0.121	0.137	0.115	0.135
30	BS	0.005	**0.003**	0.030	0.103	0.084	0.053	0.008	**0.005**	0.085	0.158	0.230	0.122	0.111	**0.004**	0.026	0.098	0.083	0.070
	sen	0.874	0.925	0.797	0.697	**0.945**	0.846	0.810	**0.872**	0.539	0.368	0.000	0.749	0.878	0.917	0.786	0.805	**0.939**	0.901
